# M2 macrophage polarisation is associated with alveolar formation during postnatal lung development

**DOI:** 10.1186/1465-9921-14-41

**Published:** 2013-04-05

**Authors:** Christina V Jones, Timothy M Williams, Kenneth A Walker, Hayley Dickinson, Samy Sakkal, Bree A Rumballe, Melissa H Little, Graham Jenkin, Sharon D Ricardo

**Affiliations:** 1 Department of Anatomy and Developmental Biology, Monash University, Clayton, Victoria, Australia; 2 The Ritchie Centre, Monash Institute of Medical Research, Monash University, Clayton, Victoria, Australia; 3 Institute for Molecular Bioscience, The University of Queensland, St Lucia, Brisbane, Australia

**Keywords:** Macrophage, CSF-1, Lung development, M2, Alveolarisation

## Abstract

**Background:**

Macrophages are traditionally associated with inflammation and host defence, however a greater understanding of macrophage heterogeneity is revealing their essential roles in non-immune functions such as development, homeostasis and regeneration. In organs including the brain, kidney, mammary gland and pancreas, macrophages reside in large numbers and provide essential regulatory functions that shape organ development and maturation. However, the role of macrophages in lung development and the potential implications of macrophage modulation in the promotion of lung maturation have not yet been ascertained.

**Methods:**

Embryonic day (E)12.5 mouse lungs were cultured as explants and macrophages associated with branching morphogenesis were visualised by wholemount immunofluorescence microscopy. Postnatal lung development and the correlation with macrophage number and phenotype were examined using Colony-stimulating factor-1 receptor-enhanced green fluorescent protein (*Csf1r*-EGFP) reporter mice. Structural histological examination was complemented with whole-body plethysmography assessment of postnatal lung functional maturation over time.

Flow cytometry, real-time (q)PCR and immunofluorescence microscopy were performed to characterise macrophage number, phenotype and localisation in the lung during postnatal development. To assess the impact of developmental macrophage modulation, CSF-1 was administered to neonatal mice at postnatal day (P)1, 2 and 3, and lung macrophage number and phenotype were assessed at P5. EGFP transgene expression and *in situ* hybridisation was performed to assess CSF-1R location in the developing lung.

**Results:**

Macrophages in embryonic lungs were abundant and densely located within branch points during branching morphogenesis. During postnatal development, structural and functional maturation of the lung was associated with an increase in lung macrophage number. In particular, the period of alveolarisation from P14-21 was associated with increased number of *Csf1r*-EGFP+ macrophages and upregulated expression of *Arginase 1 (Arg1), Mannose receptor 1 (Mrc1)* and *Chemokine C-C motif ligand 17 (Ccl1*7), indicative of an M2 or tissue remodelling macrophage phenotype. Administration of CSF-1 to neonatal mice increased trophic macrophages during development and was associated with increased expression of the M2-associated gene *Found in inflammatory zone (Fizz)1* and the growth regulator *Insulin-like growth factor (Igf)1*. The effects of CSF-1 were identified as macrophage-mediated, as the CSF-1R was found to be exclusively expressed on interstitial myeloid cells.

**Conclusions:**

This study identifies the presence of CSF-1R+ M2-polarised macrophages localising to sites of branching morphogenesis and increasing in number during the alveolarisation stage of normal lung development. Improved understanding of the role of macrophages in lung developmental regulation has clinical relevance for addressing neonatal inflammatory perturbation of development and highlights macrophage modulation as a potential intervention to promote lung development.

## Background

A diverse network of regulators govern the developmental transformation from multipotent progenitors in the post-induction lung buds to the complex architecture and highly specialised terminal cell types that make up the mature lung. These include a range of growth factors, signalling pathways and transcriptional regulators that arise from epithelial, mesodermal and mesothelial origins [Reviewed in [1]]. Another important component of the lung organogenic milieu is the tissue macrophage. Traditionally associated with host defence, inflammation and scavenging functions, a greater appreciation of macrophage diversity has revealed broader functions of macrophages including vital roles in tissue repair [2-6] and organ development [7-11].

Macrophages first arise in the yolk sac around embryonic day (E)8 in the mouse, and migrate into the developing head before colonising the entire embryo [12-14]. Large numbers of macrophages are present in virtually all developing organs, with maximum numbers correlating with key periods of organogenesis [15]. Macrophages contribute to development through apoptosis, phagocytic clearance of cellular debris associated with tissue remodelling, and as potent effector cells producing a range of trophic factors that stimulate growth, regulate cellular differentiation and promote angiogenesis [Reviewed in [16]]. Furthermore, mice deficient in tissue macrophages display a range of developmental abnormalities including skeletal and neurological deficiencies and impaired growth and fertility [17-19].

Macrophages are essential in the normal development of the mammary gland, pancreas and kidney; organs which, similar to the lung, develop through branching morphogenesis. Normally, macrophages are located surrounding developing terminal buds but, in their absence, branching is impaired resulting in atrophic, poorly-branched terminal buds in the mammary gland [9,20], and abnormal islet cell morphology and reduced insulin production in the pancreas [21,22]. Furthermore, the addition of the key macrophage regulatory cytokine colony-stimulating factor (CSF)-1 to embryonic organ cultures was shown to enhance development of the pancreas [8] and kidney [11], which was associated with increased number of tissue macrophages. While the organogenic contribution of macrophages to these organs is well described, less has been investigated regarding their roles in the development of the lung.

Macrophages are present in the lung from the initiation of development, and at E10 are located abundantly in the mesenchyme and in association with elongating lung buds [23,24]. Fetal lung macrophages likely contribute to lung development through the regulation of apoptosis and clearance of cellular debris. Defective pulmonary phagocytosis in the phosphatidylerine receptor (*psr)*^−/−^ mutant mouse is associated with impaired removal of apoptotic cells during development, which in turn results in solid lungs devoid of alveoli [25]. Macrophages in the lung are also sources of trophic factors such as insulin-like growth factor (IGF)-1 [26] and wingless-type MMTV integration site (Wnt)7b [27], both of which are important regulators in lung development.

To date, the understanding of lung macrophage function has focussed on pathological implications in settings associated with neonatal inflammation with little insight regarding their contribution to normal developmental regulation. In this study, we provide the first report characterising macrophages during the alveolarisation stage of lung development in the mouse. Macrophages in the postnatal lung displayed a phenotype indicative of an M2 or alternatively activated macrophage polarisation state, which is characteristic of macrophages involved in trophic and tissue remodelling functions. Furthermore, the number of CSF-1 receptor (CSF-1R)+F4/80+ macrophages was increased during alveolarisation and, together with the expression of M2-associated genes, indicates the importance of trophic macrophages during this period of significant tissue remodelling.

## Methods

### Animals

All animal experiments were approved in advance by the Monash University Animal Ethics Committee and conducted in accordance with the “Australian Code of Practice for the Care and Use of Animals for Scientific Purposes” (7^th^ Edition, 2004). For embryonic lung culture, time-mated C57BL/6J females were humanely euthanised by cervical dislocation at 12.5 days postcoitum, with 0.5 defined as noon on the day a plug was detected. Embryos were collected, development was assessed using the Theiler Staging (TS) criteria (TS 15–16/27–31 somites) and the lungs were dissected. Postnatal lung analyses were performed on *Csf1r*-EGFP mice, which directs enhanced green fluorescent protein (EGFP) expression to cells of the myeloid lineage under the control of the *Csf1r* promoter [14]. Neonatal mice were administered mouse recombinant CSF-1 (1 μg/g bodyweight; University of Queensland Protein Facility, Brisbane, Australia) in phosphate buffered saline (PBS) via intraperitoneal (i.p.) injection at a final volume of 50 μl at postnatal day (P)1, 2 and 3, with P1 defined as day of birth [2]. Littermate controls received vehicle PBS at the equivalent final volume.

### Embryonic lung culture and wholemount immunofluorescence labelling

Embryonic lungs were transferred onto polycarbonate membranes (3 μm pore size; GE Water and Processing Technologies, Oakville, Canada), floating on serum-free media in a 24 well plate (BD Biosciences). Culture media was composed of Dulbecco’s Modified Eagle Medium F/12 (Gibco/Invitrogen, Mulgrave, VIC, Australia), supplemented with 2.5 mM L-glutamine (Gibco/Invitrogen), 5 μg/ml insulin transferrin selenium (Gibco/Invitrogen) and 100 μg/ml penicillin streptomycin (Gibco/Invitrogen). Organs were incubated for 48 hours at 37°C in 5% CO_2_. Explants were fixed in ice-cold methanol (for 30 min at −20°C) and wholemount immunolabelled to visualise macrophages in development. Explants were permeablised in 0.1% Triton X (in PBS for 10 min) and non-specific binding was blocked by incubation with 10% goat serum and 2% bovine serum albumin (BSA; in PBS for 30 min). Explants were incubated with rat anti-F4/80 (1:100; Serotec, Kidlington, UK; Clone Cl:A3-1) and rabbit anti-E-cadherin (1:100: Cell Signalling Technologies, Danvers, MA, USA; Clone 24E10) primary antibodies (at 37°C for 2 hrs) to demarcate macrophages and lung epithelium, respectively. Explants were washed in PBS (3× 5 min at room temperature), incubated with Alexa Fluor® goat anti-rat 555 and goat anti-rabbit 488 (Invitrogen; 1:500) secondary antibodies (at 37°C for 1 hr). Membrane-bound explants were placed on glass slides with PBS and coverslipped.

### Postnatal lung histology and macrophage immunofluorescence labelling

Lungs were reinflated and fixed *in situ* through intratracheal instillation of 10% buffered formalin at a pressure of 20 cmH_2_0. After ligating the trachea, the entire thorax was immersion fixed for 24 hr before lungs were dissected. To assess histology, lungs were processed, embedded in paraffin wax, sectioned at 5 μm, mounted on Polylysine™ slides (Menzel-Glaser, Braunschweig, Germany) and stained with haematoxylin and eosin. For immunofluorescence labelling, excised lungs were placed in 30% sucrose solution (in PBS) and allowed to infiltrate overnight at 4°C. Organs were immersed in OCT compound (Sakura, Torrance, CA, USA) in Tissue-Tek® cryomoulds (Sakura) and frozen by floating moulds on chilled isopentane on dry ice. Lungs were cryosectioned at 5 μm and mounted on SuperFrost® Plus slides (Menzel-Glaser). For macrophage visualisation, sections were blocked in 10% goat serum, incubated with rat anti-F4/80 primary antibody (1:100; Serotec), washed and incubated with AlexaFluor® goat anti-rat 488 secondary antibody (1:500; Invitrogen). Sections were counterstained with DAPI nuclear stain (1:10,000 in PBS; Invitrogen) for 5 minutes, washed, mounted with DAKO fluorescent mounting medium (DAKO Cytomation, Botany, NSW, Australia) and coverslipped.

### Flow cytometry

Whole lungs underwent enzymatic and mechanical digestion to yield a single cell suspension as described previously [2]. In brief, organs were finely minced and incubated in 1 ml digestion buffer; comprising 1 mg/ml collagenase/dispase (Roche Diagnostics, Indianapolis, IN, USA), 0.1% DNase I (Roche Diagnostics) and 5 mM CaCl2 in Hank’s Balanced Salt Solution (Invitrogen) at 37°C for 20 minutes. Lungs were mechanically disrupted using a 1000 μl pipette, before cells were gently passed through a 25-gauge needle to yield a single cell suspension. Cell suspensions were washed in fluorescence-activated cell sorting (FACS) buffer; comprising PBS supplemented with 0.2% BSA, 0.5 M ethylenediaminetetraacetic acid (EDTA) and 0.02% sodium azide, and centrifuged at 485 relative centrifugal force (rcf; for 5 minutes at 4°C). Red blood cells were lysed by resuspending samples in 1 ml of red blood cell lysis buffer (at 37°C for 1 min; 8.3 g/L ammonium chloride; pH 7.5;) and cell suspensions were filtered through a 40 μm cell strainer (BD Biosciences, North Ryde, NSW, Australia). Cell counts were performed using a Coulter® Particle Count and Size Analyzer (Beckman Coulter Australia Pty Ltd, Gladesville, NSW, Australia). To assess macrophages across postnatal development, 1×10^6^ cells were immunolabelled with anti-CD45 PE Cy5-conjugated antibody (1:1000; BD Biosciences; Clone 30-F11) at a final volume of 20 μl for 20 minutes at 4°C in a 96 well plate. Cells were washed in FACS buffer and centrifuged, repeated twice, before being resuspended in 200 μl FACS buffer and run on a BD FACSCalibur cytometer (BD Biosciences). To assess macrophages at P5 following CSF-1 administration, cells were immunolabelled with anti-CD45 APC Cy7-conjugated (1:800; BioLegend, San Diego, CA, USA; Clone 30-F11) and rat anti-F4/80 APC-conjugated (1:200; eBioscience, San Diego, CA, USA; Clone BM8) antibodies. Samples were run on a BD FACSCanto II cytometer (BD Biosciences). Data analysis was performed using Flow Jo FCS analysis software (Tree Star Inc., Ashland, OR, USA).

### Plethysmography

Respiratory physiology across a time course of postnatal development was assessed using unrestrained barometric whole-body plethysmography, as described previously [28,29]. In brief, mice were placed in a sealed cylindrical Perspex chamber (Neonate; 75 mm × 50 mm, Adolescent/Adult; 150 mm × 50 mm), where changes in pressure caused by breath tidal movements were measured using a volumetric pressure transducer (model PT5A; Grass Instrument Co., Quincy, MA, USA), amplified (Octal Bridge Amp model ML228 and Powerlab 8/30 model ML870; ADInstruments, Bella Vista, NSW, Australia) and the respiratory trace patterns recorded using Chart™ software (v5.1; ADInstruments). At the beginning of each session the plethysmograph was calibrated by measuring the pressure deflection caused by the injection of a known volume (300 μl) of air into the chamber. The temperature and relative humidity within the chamber were noted at the beginning and end of recordings (model HM34; Vaisala, Hawthorn, VIC, Australia). Waveform analysis (Chart™; ADInstruments) of respiratory traces was used to directly derive the pressure deflection per tidal breath (P_T_), total breath cycle time (T_tot_; sec), breath frequency (f; breaths/min), inspiration time (T_i_; sec) and expiration time (T_e_; sec). To calculate tidal volume (V_T_; mL), the P_T_ value obtained from the respiratory trace was inputted into the equation of Drorbaugh and Fenn [30], which was subsequently used to determine minute volume (V_E_; mL/min; V_T_ × f) and inspiratory flow rate (V_T_/T_i_; mL/sec).

### QPCR

Semi-quantitative real-time (qPCR) was used to assess gene expression in whole lungs across postnatal development and in response to CSF-1. Lungs were dissected and snap frozen in RNAlater® RNA stabilisation reagent (Qiagen, Doncaster, VIC, Australia). Total RNA was extracted from organs using an RNeasy Mini Kit (Qiagen) and concentration and purity were analysed using a Nanodrop® Spectrometer (Nanodrop® Technologies, Wilmington, DE, USA). RNA was converted to cDNA using a High Capacity cDNA Reverse Transcription Kit (Applied Biosystems, Mulgrave, VIC, Australia). qPCR was performed using Taqman® Gene Expression Assays (Applied Biosystems) which provided pre-designed primer and probes to assess the genes *β-actin* (*Actb*; Assay ID: Mm00607939_s1), *Chemokine C-C motif ligand (Ccl)2* (Mm00441242_m1), *Inducible nitric oxide synthase* (*Nos2*; Mm00440485_m1), *Tumor necrosis factor-α* (*Tnf*; Mm00443258_m1), *Ccl17* (Mm00516136_m1), *Arginase 1* (*Arg1*; Mm00475988_m1), *Igf1 (*Mm00439561_m1) and *Found in Inflammatory Zone 1* (*Fizz1;* Mm00445109_m1). Reactions were performed in triplicate and run on a 7500 Real-Time PCR machine using SDS Software (v1.3; Applied Biosystems). Threshold cycle (Ct) values were normalised against endogenous *Actb* expression and presented as relative quantification (RQ).

### *In situ* hybridisation and microscopy

Section *in situ* hybridisation for the *Csf1r* gene was performed on paraffin-embedded, 5 μm sections of E12.5 embryonic lungs, as described previously [31] (Probe ID: MGI:50000914; http://www.gudmap.org). Sections were counterstained with haematoxylin. Light and fluorescence microscopy were performed using an Olympus Provis AX70 microscope (Olympus, Mt Waverley, VIC, Australia) and AnalysisB software (Soft Imaging Systems GmbH, Muenster, Germany). Bright field images were captured using a DP70 colour camera (Olympus). Fluorescence images were captured using an F-View black and white camera (Olympus). Image preparation and compilation was performed using AnalysisB software (Soft Imaging Systems) and Microsoft Power Point (Microsoft Corporation, Redmond, WA, USA).

### Statistical analysis

Data is presented as mean ± standard error of the mean (SEM). Statistical analysis was performed using GraphPad Prism™ (Version 5 for Windows; GraphPad Software Inc, La Jolla, CA, USA). Significance was assessed using a one-way ANOVA and Tukey’s post hoc test for comparisons across multiple time points or unpaired Student’s *t*-test for comparisons between two experimental groups. A p value <0.05 was considered statistically significant.

## Results

### Macrophages are abundant in embryonic lungs and localise within branch points

**Figure 1:**
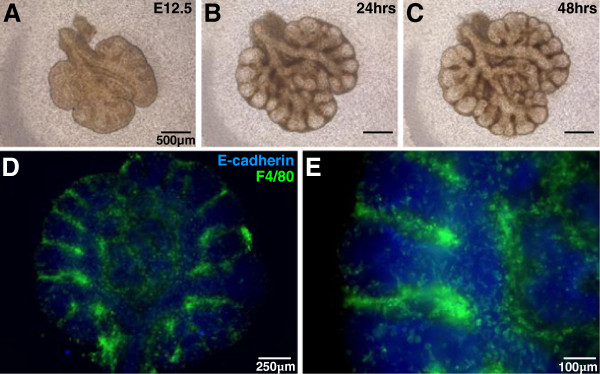
**Macrophages are abundant in developing embryonic lungs.***Ex vivo* culture of E12.5 embryonic lungs, maintained for 48 hours on floating polycarbonate membranes at the air-liquid interface, supported continuation of branching morphogenesis (**A-C**). Wholemount immunofluorescence labelling of the lung epithelium (anti-E-cadherin; blue) and macrophages (anti-F4/80; green) revealed extensive macrophage accumulation within developing lungs (**D**), and in particular within branch points **(E).**

To assess macrophages and their involvement in lung branching morphogenesis, E12.5 lungs were cultured as embryonic explants, with a continuation of branching morphogenesis observed over 48 hours of culture (Figure 1A-C). In this model system, flattening of the organ facilitated wholemount visualisation and the examination of macrophage localisation within the embryonic lung. Immunofluorescence labelling demonstrates that embryonic macrophages express the mature macrophage marker F4/80 and are found abundantly within embryonic lungs undergoing branching morphogenesis (Figure 1D&E). In particular, the dense concentration of macrophages localised within branch points is prominent (Figure 1E). Such abundance, branch-specific location and intimate epithelial interaction support the relevance of macrophages in the regulation of lung development. Furthermore, this system indicates the importance of early fetal macrophages in colonising organs undergoing development. With the explant system eliminating the contribution of infiltrating cells at later stages of development, it also indicates that the large numbers of macrophages observed are seeded within the lung before E12.5, and are then maintained through local mechanisms to support ongoing branching morphogenesis.

### Macrophages are associated with the structural and functional postnatal maturation in the mouse

In the mouse, the lung undergoes a significant period of postnatal development comprising both the closing stages of the saccular phase (E18.5–P5) and the alveolarisation phase (P5-P36) [1,32], and thereby provides an important animal species for investigating aspects of developmental regulation. The structural and functional maturation across the time course of postnatal lung development was characterised in *Csf1r*-EGFP mice.

**Figure 2:**
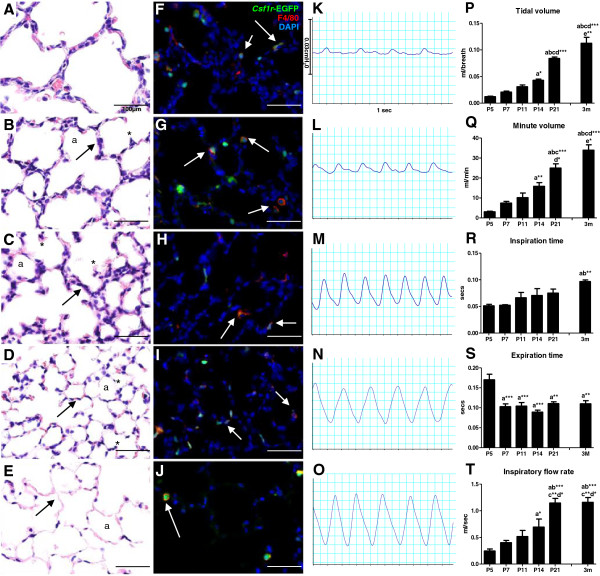
**Postnatal structural and functional lung maturation in the mouse.** Representative photomicrographs of postnatal lung histology, immunofluorescence macrophage localisation and respiratory trace patterns at P5 (**A,F,K**), P7 (**B,G,L**), P14 (**C,H,M**), P21 (**D,I,N**) and 3 months (**E,J,O**). Structural maturation (**A-E**) is demonstrated by a reduction in alveoli (a) size through the formation of new secondary septae (*) and a thinning of alveolar walls (arrows). Structural maturation accompanies functional maturation as indicated by deeper, more even respiratory trace patterns (**K-O**), which was quantified through waveform analysis (**P-T**). Postnatal lung development is accompanied by significant numbers of CSF-1R+ cells (green), which are predominantly macrophages (arrows) as indicated by F4/80 co-expression (red; **F-J**;). n=4 littermates/time point. ‘a’ represents significant difference compared to P5, ‘b’ compared to P7, ‘c’ compared to P11, ‘d’ compared to P14, ‘e’ compared to P21. Asterisks following letters represent level of significance, where *=p<0.05, **=p<0.01, ***=p<0.001.

Structural maturation facilitates the progressive gain in gas exchange efficiency; from large, thick-walled terminal sacs to smaller, thin-walled alveoli with a large surface area (Figure 2A-E). Histologically at P5, the lung parenchyma consisted of large terminal sacs (Figure 2A), and with continuing alveolarisation the subdivision into smaller alveoli through the process of secondary septation was evidenced by the formation of ridges on sac walls invading into the alveolar space (Figure 2B&C). Continued secondary septation was evident at P14, with significant numbers of smaller alveoli present (Figure 2C). By P21 there was considerable thinning of alveolar walls bringing blood vessels into close association with the epithelium lining the alveolar space (Figure 2D). In these later stages of the alveolarisation phase, much of the secondary septation was complete and maturation involved thinning of the alveolar wall interstitium (Figure 2D). The adult lung at 3M showed all the structural hallmarks of an efficient gas exchange organ; large number of alveoli providing a large surface area and extremely thin walls to allow for efficient gas exchange (Figure 2E). Postnatal development was accompanied by the identification of macrophages in the lung parenchyma. Visualised by *Csf1r*-EGFP expression, these myeloid cells were predominantly macrophages, evident by their consistent co-expression of F4/80 (Figure 2F-J). The large numbers of macrophages within the lung from P5-P21 correlates with the key period of alveolar development.

These structural changes were mirrored by the functional improvements observed during postnatal development. Trace recordings from unrestrained barometric whole-body plethysmography illustrated the breath patterns and functional maturation of the lungs of mice during postnatal development through measurement of tidal pressure changes within the chamber due to respiration (Figure 2K-0). At P5, before alveolarisation, the respiratory capacity was limited, evidenced by trace recordings where breaths were shallow, uneven and dispersed (Figure 2K). With maturation and the formation of increasing numbers of alveoli from P7-P14 (Figure 2L&M), breaths became deeper, more frequent and more even. At P21, a breath pattern comparable to the adult was observed (Figure 2N), however as lung growth continued tidal volume increased, as evidenced by the increased amplitude of the trace pattern of the 3M lung (Figure 2O). Waveform analysis provided a quantitative assessment of lung function parameters to provide a functional correlation with the structural maturation of the postnatal lung. Progressive increases in tidal and minute volume were evident during postnatal lung development, increasing 8-fold (0.012 ± 0.001 vs. 0.113 ± 0.011, p<0.001; Figure 2P) and 10-fold (3.09 ± 0.30 vs. 33.93 ± 2.70, p<0.001; Figure 2Q) from P5 to 3M, respectively, as the number of alveoli and overall size of the gas exchange compartment of the lung increased. As observed in the respiratory trace, breath patterns in immature lungs were quite uneven with short inhalations and slow dribbled exhalations at P5 and P7. This was also demonstrated in the waveform measurements, where expiration time decreased with the onset of alveolarisation (Figure 2S) and inspiration time progressively increased (Figure 2R) as a more even breath pattern emerged. A progressive increase in inspiratory flow rate was also observed, increasing approximately 4-fold from P5-P21 (0.24 ± 0.04 vs. 1.15 ± 0.09, p<0.001) when a maximum flow rate was then reached and maintained in the adult lung at 3M (Figure 2T).

### Lung macrophage number is increased during alveolarisation

**Figure 3:**
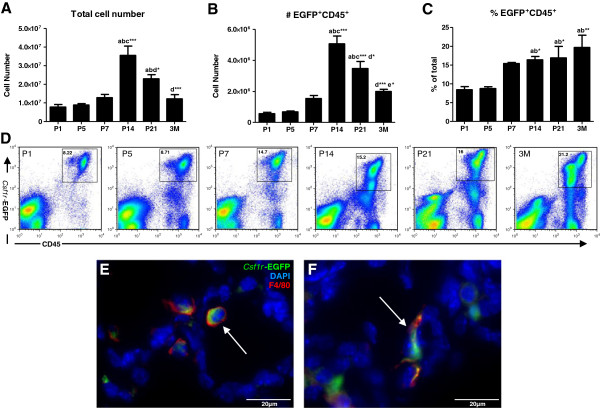
**Macrophages are increased during alveolarisation in the developing mouse lung.** Flow cytometric analysis of CSF-1R+ leukocyte (*Csf1r-*EGFP+CD45+) number (**B**) and proportion (**C**) within whole lungs (**A**) across a timecourse of postnatal development. Representative plots displaying gating of EGFP and CD45 double positive cells at each time point (**D**). n=4-5 lungs/time point. ‘a’ represents significant difference compared to P1, ‘b’ compared to P5 ‘c’ compared to P7, ‘d’ compared to P14, ‘e’ compared to P21. Asterisks following letters represent level of significance, where *=p<0.05, **=p<0.01, ***=p<0.001. *Csf1r*-EGFP+ cell localisation (green) in the developing lung at P7 which were confirmed as predominantly macrophages by F4/80 immunolabelling (red), and were composed of both alveolar (**E**) and interstitial (**F**) macrophage subpopulations (arrows).

To more comprehensively assess the correlation between macrophages and alveolarisation, flow cytometry was performed to quantitatively examine the proportion and number of macrophages in the lung during postnatal development and into adulthood (Figure 3A-C). Again EGFP transgene expression facilitated the quantification of *Csf1r*+ myeloid cells (Figure 3D), which were confirmed as predominantly macrophages by consistent co-expression of F4/80 (Figure 2F-J) evident in both alveolar and interstitial macrophage subpopulations (Figure 3E&F). Lungs were analysed at P1 in the saccular stage, at P5 when the lung transitions from the saccular to alveolarisation stage, at P7, P14 and P21 during alveolarisation, and at 3M in the mature lung. During postnatal life the overall cellularity of the lung gradually increased peaking at P14 during alveolarisation with an approximately 5-fold increase compared to P1 (7.78 ± 0.14 ×10^6^ vs. 35.50 ± 5.01 ×10^6^, p<0.001; Figure 3A). From P14 onwards, as alveoli continued to develop and mature the total cellularity of the lung decreased. At 3M, despite the overall size difference, cellularity was comparable between the mature lung composed primarily of air sacs and the dense lung at P1 (7.78 ± 0.14 ×10^6^ vs. 12.24 ± 2.23 ×10^6^, p=ns). Similarly, the number of macrophages followed a parallel trend, and increased 9-fold from P1 to P14 (5.65 ± 0.87 ×10^5^ vs. 50.68 ± 4.95 ×10^5^, p<0.001), before a −2.5-fold reduction by 3M (50.68 ± 4.95 ×10^5^ vs. 19.93 ± 1.39 ×10^5^, p>0.05; Figure 3B). In contrast to number, the proportion of macrophages in the lung steadily increased throughout postnatal life (Figure 3C). During the saccular stage at P1 and P5, macrophages represented approximately 8% of cells in the lung. This is significantly increased at P14 and P21 during alveolarisation to approximately 16% (p<0.05), and at 3M a significant resident macrophage population was maintained in the adult lung (Figure 3C).

### Macrophages are polarised to an M2 phenotype during alveolarisation

**Figure 4:**
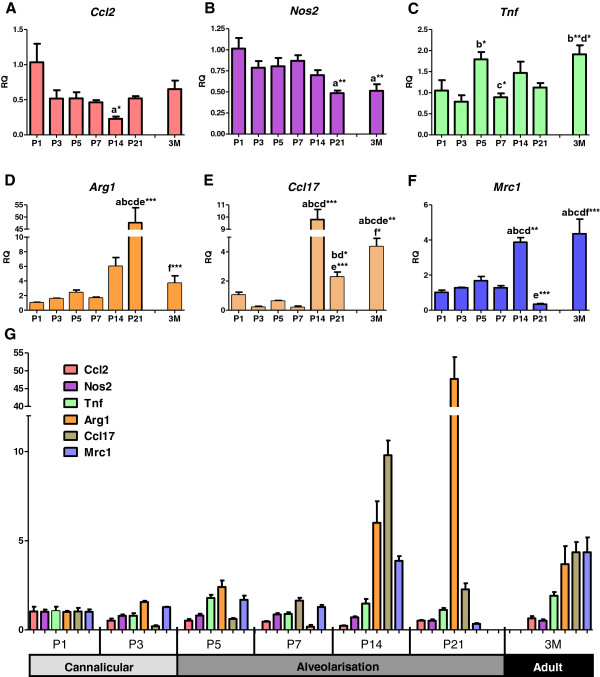
**Alveolar development is associated with M2 macrophage polarisation.** qPCR assessment of gene expression indicative of different macrophage polarisation states. Expression of pro-inflammatory M1 (**A-C**) and remodelling M2 (**D-F**) genes were normalised against *β-actin* expression and presented as relative quantification (RQ) compared to P1. All genes displayed on the same axis demonstrate the upregulation in M2 markers during alveolarisation (**G**). n=4 littermate lungs/time point. ‘a’ represents significant difference compared to P1, ‘b’ compared to P3, ‘c’ compared to P5, ‘d’ compared to P7. ‘e’ compared to P14, ‘f’ compared to P21. Asterisks following letters represent level of significance, where *=p<0.05, **=p<0.01, ***=p<0.01.

Correlations between macrophage phenotype and stages of lung development were investigated by analysing expression of genes indicative of different macrophage activation states (Figure 4). An upregulated expression of *Ccl2*, *Nos2* and *Tnf* is associated with an M1 or classical phenotype where macrophages contribute to host defence. M2 or alternatively activated macrophages are important in tissue remodelling, immunoregulatory and trophic functions, and are characterised by upregulation of genes including *Arg1*, *Ccl17*, *Mrc1*. A limited correlation between M1 gene expression and postnatal lung development was observed, although *Ccl2* expression was highest after birth (Figure 4A) and *Nos2* decreased in later life at P21 and 3M (Figure 4B). In contrast, significant upregulation of M2 genes showed a distinct correlation with the key period of alveolar development. *Arg1* expression was low in early postnatal life and began to increase at P14 (Figure 4D). At P21, *Arg1* expression peaked and was 48-fold higher than at P1 (p<0.01), before returning to a low level of expression in the adult lung. *Ccl17* was also shown to increase during the alveolarisation stage (Figure 4E). After low expression from birth to P7, *Ccl17* expression increased 9-fold at P14 (p<0.001) before decreasing by P21. The resident population of macrophages in the mature lung at 3M also maintained significant *Ccl17* expression. Similarly, *Mrc1* expression remained unchanged throughout early postnatal lung development but peaked at P14 with a 3-fold increase compared to P7 (p<0.01; Figure 4F). Expression was decreased at P21 before high levels of *Mrc1* expression were maintained in the resident lung macrophage population at 3M. When all the genes analysed are presented on the same graph, the increase in the three genes indicative of an M2 macrophage phenotype is particularly evident (Figure 4G). This demonstrates a clear correlation between M2 macrophage phenotype and the key period of alveolarisation.

### CSF-1 administration increases developmental macrophages in the lung and is associated with increased *Igf*1 expression

**Figure 5:**
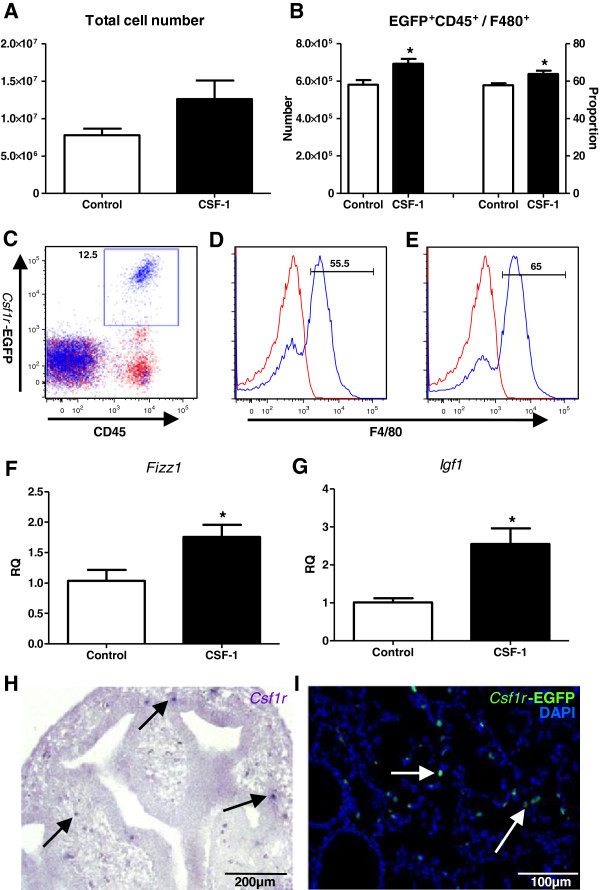
**CSF-1 increases developmental macrophages and promotes M2 gene and IGF-1 upregulation.** Flow cytometric analysis of macrophages (**A-E**) and qPCR analysis of gene expression in whole lungs (**F**&**G**) at P5, following administration of mrCSF-1 (1 μg/g i.p. at final volume of 50μl; black bars) or PBS vehicle control (white bars) to littermate neonatal *Csf1r*-EGFP mice at P1, 2 and 3. Cells from whole lungs (**A**) were gated on *Csf1r-*EGFP+CD45+ myeloid cells (**C**) and further gated on F4/80 expression to assess macrophage number and proportion (**B**), as displayed in representative histograms from control (**D**) and CSF-1-treated mice (**E**). Staining (blue) is overlayed with an isotype control (red). *Igf1* (**G**) and M2 gene *Fizz1* (**F**) expression was normalised against *β-actin* expression and presented as RQ compared to controls. n=3-4 littermate lungs/treatment. *=p<0.05. Photomicrographs of *in situ* hybridisation for a *Csf1r* riboprobe at E12.5 **(H**; purple) and fluorescence *Csf1r-*EGFP transgene expression at P5 **(I**; green) demonstrated that during development the CSF-1R is expressed on interstitial myeloid cells (arrows), and not on the developing lung epithelium.

CSF-1 is the primary regulator of macrophage differentiation, survival and proliferation, and during development it plays an essential and non-redundant role in regulating organogenic macrophage functions [17,18,33,34]. Administration of CSF-1 to neonatal mice was shown to increase the number and proportion of developmental macrophages within the lung at P5. Flow cytometric analysis was performed on whole lungs, with populations of *Csf1r*-EGFP+ leukocytes (Figure 5C) further gated on F4/80 expression to investigate macrophages (Figure 5D&E). There was a trend towards an increase in total cellularity in CSF-1-treated lungs (Figure 5A). CSF-1 treatment resulted in a 6% increase in macrophage number (58.10 ± 2.49 ×10^4^ vs. 69.32 ± 2.56 ×10^4^, p<0.05), and a 19% increase in macrophage proportion (57.77 ± 1.15% vs. 63.88 ± 1.78%, p<0.05), compared to PBS-treated littermates (Figure 5B). Analysis of gene expression also indicated that the CSF-1-mediated increase in macrophages was associated with upregulation of the Th2-associated molecule *Fizz1* (1.05 ± 0.18 vs. 1.56 ± 0.05, p<0.05; Figure 5F) and the important growth regulator *Igf1* (1.01 ± 0.11 vs. 2.55 ± 0.41, p<0.05; Figure 5G).

These effects of CSF-1 administration were confirmed to be via a macrophage-mediated mechanism as the CSF-1R was expressed exclusively on interstitial myeloid cells and not other cells of the developing lung. *In situ* hybridisation for the CSF-1R at E12.5 (Figure 5H) and *Csf1r* driven EGFP transgene expression at P7 (Figure 5I) confirmed that the trophic activity of CSF-1 during lung development is through macrophage regulation. Furthermore, the finding that CSF-1 supplementation promoted a trophic M2 macrophage phenotype highlights CSF-1 and the manipulation of CSF-1-responsive cells as a potential intervention for rescuing or promoting organ development and maturation.

## Discussion

The renewed interest in macrophages has stemmed from an increased understanding of monocyte/macrophage heterogeneity and how it relates to functional diversity [35-38]. Differential activation states have been broadly classified as M1, which encompasses macrophages involved in host defence and inflammation, and M2, which represent a more wound healing or tissue remodelling phenotype. Despite the M1/M2 activation dichotomy arising from studies of tissue disease and repair, understanding macrophage phenotype and function has implications for discerning and potentially enhancing their contribution to organ development. Functions of M2 macrophages, such as extracellular matrix (ECM) production, release of trophic factors and promotion of angiogenesis, are fundamental to organogenesis. Furthermore, our microarray expression profiling has revealed that embryonic macrophages in developing lungs, kidneys and brains show a comparable gene expression profile consistent with an M2 activation state [11]. In addition, CSF-1 can also promote an ‘M2’ macrophage activation state, which is increasingly being linked to tissue repair and regeneration [2,39,40].

During embryonic development, macrophages are located abundantly within the embryo and are present in virtually all developing organs [7]. Macrophage functions that support organogenesis include clearance of apoptotic cellular debris associated with tissue remodelling [41] and the provision of trophic support by producing a range of regulatory mediators [5,42]. Macrophages also contribute to appropriate cellular differentiation [8,21,22] and angiogenic regulation [43], through both the production of angiogenic factors [44] and by physically directing angiogenic positioning [10]. As the lung buds form, macrophages surround the elongating primary bronchi [24]. The use of the *Csf1r*-EGFP reporter mice has been an important tool in demarcating the developmental role of macrophages in the embryo and has demonstrated a significant population of CSF-1R-expressing macrophages within the lungs at E13.5 [14]. Eliciting its effect through binding with the CSF-1R [45], CSF-1 is a pleiotropic growth factor also important in the regulation of pregnancy, fetal development and tissue regeneration [Reviewed in [33,34,40]].

Macrophages in the lung have been well described for their functions in host defence and inflammatory diseases, however the importance of CSF-1R+ macrophages in contributing to lung development has not been elucidated. The present study demonstrates the localisation of CSF-1R+EGFP+ alveolar and interstitial macrophages, which co-express the mature macrophage marker F4/80, in developing lungs during postnatal development. In addition, CSF-1R+ macrophages were identified in embryonic lung explants using wholemount immunofluorescence microscopy, where they were found to accumulate at branch points during lung branching morphogenesis.

The proposal that key organogenic periods are accompanied by an M2 macrophage phenotype was examined in the postnatal lung. During alveolarisation from P14 to P21, the expression of the M2 markers examined (*Arg1, Ccl17* and *Mrc1*) showed a significantly increased expression. The remodelling functions of M2 macrophages are in accordance with the structural changes occurring within the lung at this time. *Mrc1* provides an important mechanism for cellular clearance associated with homeostasis and tissue reorganisation [46]. *Arg1* is associated with collagen formation and ECM production [47]. This study thus highlights the importance of macrophages in the alveolarisation stage of lung development, and in particular the association with an M2 activation state. Furthermore in the adult lung, an upregulation of M2 genes was also observed, supporting the homeostatic and immunomodulatory functions of resident pulmonary macrophages.

We have previously reported that CSF-1-responsive developmental macrophages are associated with growth and organ development, with delivery of recombinant protein to neonatal mice resulting in increased body and organ weight [2]. The present study showed that administration of CSF-1 to neonatal mice was also increased the number of macrophages in the developing lung and promoted an increase in *Fizz1 (Retnla)* and *Igf1* expression. Fizz1 is an important mediator of lung development and maturation, and is upregulated during the saccular and alveolar stages, where its angiogenic and proliferative functions are suggested to promote alveolar development [48]. Fizz1 is also reported to participate in lung maturation by modulating surfactant production [49]. Expressed on lung cells such as mesenchymal and alveolar type II cells, the function of macrophage-derived Fizz1 is under-examined in previous reports, and its upregulation with CSF-1 supplementation indicates it may provide beneficial effects in regulating lung development.

The increase in the key growth regulator IGF-1 in response to CSF-1 administration provides insight regarding the potential mechanism of trophic macrophage function in organogenesis, and also supports an emerging link between CSF-1, macrophages and the IGF-1 growth axis [34]. Interestingly, many of the growth and developmental deficiencies observed in CSF-1-deficient mice are common to IGF-1-deficient animals [50]. Moreover, an interaction between CSF-1 and the IGF-1 growth axis is supported by the finding that CSF-1-deficient rats fail to produce the postnatal spike in IGF-1 [34]. Furthermore, IGF-1 production as a key mechanism of trophic macrophage function is supported by a previous study which demonstrated that kidney regeneration in an experimental model of acute kidney disease is mediated by CSF-1-responsive macrophages and an upregulation of IGF-1 [2]. This has important parallels for normal lung development as IGF-1 increases during alveolarisation, and the promotion of lung maturation using retinoic acid and dexamethasone has been shown to correlate positively with increased levels of IGF-1 [51].

Identification of M2-polarised macrophages as an important component of the organogenic milieu during alveolar development has important potential clinical implications, not only for understanding normal developmental processes, but also for addressing lung immaturity and the impact of neonatal inflammation of developmental perturbation. Inflammatory activation of macrophages not only contributes to tissue damage and perturbation of organ development through pro-inflammatory injury, but also disrupts morphogenesis in the lung and alters the expression of key genes important in lung development [23,52]. Nuclear factor kappa-light-chain-enhancer of activated B cells (NF-κB) signalling in fetal macrophages upregulates pro-inflammatory mediators such as interleukin-1β and alters expression of Wnt7b, bone morphogenic protein-4 [23] and fibroblast growth factor-10 [52]. Given the correlation between the timing of lung structural establishment and lung immunological maturity [53], it is plausible that the developmental deficits associated with inflammation may also result from skewing of macrophages prematurely away from their organogenic activities toward pro-inflammatory mediation roles.

Understanding the regulation of alveolar development has particular relevance with regard to clinical implications of developmental perturbation, and especially in the setting of preterm birth. The neonatal mouse provides an excellent model for studying these aspects of lung development as mice are born at a stage where earlier aspects of organ development are still ongoing. The period of postnatal development in the mouse was characterised; correlating structural and functional maturation with macrophage localisation. The histological time course of postnatal lung development demonstrates the processes of structural maturation, whereby large saccules evident at the end of the saccular stage at P5 begin to subdivide through secondary septation to form definitive alveoli clearly evident at P21. This period of alveolarisation is critical in establishment of the gas exchange units required for proper function of the lung. This is evidenced by the chronic lung dysfunction associated with disruptions of alveolar development that often result from preterm birth and injurious therapeutic interventions that are required to keep the neonate alive [54,55]. Indeed, Mund et al. reported that murine alveolarisation occurs in two stages; phase 1 from P4-21 where alveoli arise from immature septae, and phase 2 from P21-36 where alveoli lift off from existing mature alveoli [32].

The histological time course of postnatal lung development was complemented by analysis of functional maturation over this time. Changes in breath cycle parameters were examined throughout postnatal lung development using unrestrained barometric whole-body plethysmography. This technique has been utilised in adult models of lung injury [28,29] and was optimised for use in neonatal mice in our study. Modifications including the adjustment of the Perspex chamber size and increased sensitivity settings for pressure transduction recording enabled the measurement of lung function from as early as P5. Trace recordings showed discernable changes in breath patterns from P5 to adulthood, which when analysed provided quantitative changes in lung function. As the number of alveoli increased and the gas exchange compartment expanded, there was a correlation with changes in lung function parameters including an 8- and 10-fold increase in tidal and minute volume from the beginning of alveolarisation at P5 to adulthood. Normalisation of inspiratory flow rate and expiratory time were also observed over this time period of postnatal development as the chest wall and diaphragm mature.

The unique saccular architecture of the mature lung is associated with a different developmental cellularity pattern than that of solid organs. Overall cellularity increases rapidly in the alveolarisation phase and peaks at P14. From this stage, development is associated with significant remodelling and apoptosis as alveolar sacs form and mature, resulting in a decrease in cellularity in the adult mature lung. By digesting whole lungs, both interstitial as well as alveolar macrophages were able to be included for analysis, as opposed to the commonly used bronchoalveolar lavage-based collection method which restricts analysis to alveolar macrophages only. The proportion of macrophages in the lung is highest during the alveolarisation stage of lung development. A significant resident population is also maintained in the adult lung post completion of development, indicative of the unique air-exposed environment and the importance of pulmonary macrophages in clearing inhaled debris and modulating immune responses. The increased proportion of macrophages observed during alveolarisation – a time that lacks immunological relevance in this normal setting - therefore suggests that macrophages are associated with developmental functions.

## Conclusion

This study has demonstrated that macrophages provide a valuable contribution to normal lung development, and in particular that macrophages are increased and display an M2 polarisation phenotype during alveolarisation. An improved understanding of the organogenic environment important in regulating alveolar development has significant clinical relevance. The impact of inflammation and therapeutics on organogenic macrophage populations should be considered when studying the dysregulation and damage of the neonatal lung associated with preterm birth. It also supports research into modulation of macrophages in lung development to provide a novel intervention for enhancing lung maturation.

## Competing interests

CVJ & SDR hold a patent relating to the content of this manuscript

## Authors’ contributions

CVJ contributed to study design, performed the acquisition and analysis of data, and wrote the manuscript. TMW and SS assisted with flow cytometry, KAW participated in embryonic explant culture, HD assisted with plethysmography and lung histology, BAR performed *in situ* hybridisation under the supervision of MHL, GJ provided intellectual input and critical drafting of the manuscript and SDR oversaw study design and coordination, data interpretation and writing of the manuscript. All authors read and approved the final manuscript.
